# EZH2 expression in invasive lobular carcinoma of the breast

**DOI:** 10.1186/1477-7819-11-299

**Published:** 2013-11-22

**Authors:** SiGyun Roh, Shin Young Park, Hyoung Suk Ko, Jang Sihn Sohn, Eun Jung Cha

**Affiliations:** 1 Department of Pathology, Konyang University College of Medicine and Konyang University Myunggok Medical Research Institute, 158 Gwanjeodong-ro, Seo-Gu, Daejeon 302-718, South Korea; 2 Department of Plastic & Reconstructive Surgery, Chonbuk National University School of Medicine Jeonju South Korea

**Keywords:** Breast, Enhancer of Zeste-2, EZH2, Lobular carcinoma

## Abstract

**Background:**

Invasive lobular carcinoma (ILC) is the second most common histologic type of breast cancer, but the prognosis of ILC is still controversial. Enhancer of Zeste homolog 2 (EZH2), the catalytic subunit of the Polycomb repressive complex 2 (PRC2), is frequently overexpressed in various cancers. This study evaluated the relationship between clinicopathologic characteristics and EZH2 expression.

**Methods:**

We retrospectively reviewed the medical records of 54 patients with ILC and selected 49 cases of ILC. Immunohistochemistry for EZH2 was undertaken.

**Results:**

We defined ILC as discohesive cells with a linear or nonlinear growth pattern. No statistically significant difference was found for most variables, including multifocality, menstrual status, body mass index, tumor stage (pT), lymph node stage (pN), estrogen receptor, and progesterone receptor. In contrast, nuclear grade was statistically significant and EZH2 expression was associated with high nuclear grade. In total, 80% of nuclear grade 3 cases had an EZH2 score of 4, and 86% of nuclear grade 1 cases had EZH2 scores of 1 and 2. Our cases had a score of 3 for tubule formation and a score of 1 for mitosis, and so the histologic grading consisted of grades 1 (7 cases) and 2 (42 cases) depending on the nuclear grade.

**Conclusion:**

Although EZH2 could not predict survival in our study, EZH2 expression was associated with a high nuclear grade. Most ILCs have histologic grade 2 with nuclear grade 2 or 3. Therefore, our opinion is that if ILC is diagnosed by separating the classic type and variants and considering both EZH2 expression and nuclear grade, EZH2 overexpression could help and the Nottingham grading system would be more accurate prognostic factor.

## Background

Breast cancer is the most common malignancy among women in the Western world. Invasive lobular carcinoma (ILC) was first described by Foot and Stewart [1] in 1941 and is the second most common histologic type of breast cancer, comprising 5% to 15% of newly diagnosed invasive tumors. Its incidence has been increasing over the last 20 years, mainly in women over 50 years of age, and this may be related to the use of postmenopausal hormone replacement therapy [2]. A greater tendency for multifocality and bilaterality is seen for ILC than for other mammary breast tumors. In its classic form, ILC is characterized by small, round cells that are glandular in appearance and have scant cytoplasm; these cells infiltrate the stroma in single file and surround benign breast tissues in a targeted manner [3].

Enhancer of Zeste homolog 2 (EZH2) is the catalytic subunit of the Polycomb repressive complex 2 (PRC2), which is a main histone methyltransferase that methylates lysine-27 of histone H3 [4]. EZH2 plays an essential role in epigenetic maintenance and is frequently overexpressed in a wide variety of cancerous tissue types. It is associated with poor prognosis for prostate cancer [5] and other malignancies [6,7].

The prognosis of ILC is still controversial, despite studies of prognosis that are comparable to those of stage-matched and grade-matched invasive ductal carcinoma [8]. Some studies suggest that EZH2 expression is correlated with reduced E-cadherin expression in breast cancer [9] and is an independent prediction of breast cancer recurrence and death [10,11]. Most ILCs have lost the expression of E-cadherin, whereas most other subtypes have retained expression.

In this study, we propose that all ILCs or lobular carcinomas *in situ* showed EZH2 overexpression. We investigate the association EZH2 with other clinicopathological parameters and discuss the clinical implications [11].

## Methods

### Patients

Tumor specimens were obtained from patients with ILC at Konyang University Hospital and Chonbuk University Hospital between January 2000 and December 2010. Fifty four breast tissue samples diagnosed as ILC were used. The patients had undergone lumpectomy or mastectomy and none of the patients had received radiotherapy or chemotherapy before surgery. Clinicopathologic variables, including patient age, menstrual status, body mass index, tumor size, tumor multifocality, tumor stage, and lymph node stages were used to evaluate the tumors. The histopathologic diagnoses of the tumors were described according to the WHO International Classification of Disease for Oncology. The clinical staging was determined by the TNM staging system. The histologic grade of ILCs was scored according to the Scarff-Bloom-Richardson classification. This study was approved by the Institutional Review Board (IRB) of Konyang University Hospital.

### Immunohistochemistry and interpretation

Tumor samples obtained from surgery were routinely fixed with formalin, embedded in paraffin, cut into 4 μm thick sections, and subjected to immunohistochemistry. Endogenous activity was quenched by incubation with 3% hydrogen peroxidase for 30 minutes after deparaffinization and hydration. Antigen retrieval was subsequently carried out. The primary antibody used in this investigation was EZH2 (1:200; monoclonal, BD Bioscience, San Jose, USA). Diaminobenzidine was used a chromogen, and the slides were counterstained with hematoxylin.

EZH2 expression was recorded as the percentage of epithelial cells with nuclear expression. For immunohistochemical assessment of EZH2 expression, the frequency of nuclear staining was evaluated using a semiquantitative scale: 0 = no expression; 1 = positivity in 1 to 5% = low expression; 2 = positivity in >5 to 25% = intermediate expression; 3 = positivity in >25 to 50% = high expression; and 4 = positivity in more than 50% = very high expression [7]. The arrays were independently scored by two researchers blinded to patient outcomes. Estrogen receptor and progesterone receptor levels had been determined by immunohistochemical staining with monoclonal antibodies against estrogen receptor and against progesterone receptor as part of the routine clinical evaluation. Positive hormone receptor status was defined as nuclear staining in at least 10% of invasive cancer cells.

### Statistical analysis methods

Data were analyzed using the Statistical Package for the Social Science Version 17.0 (SPSS 17.0). Pearson’s chi-square test was used to evaluate associations between EZH2 expression and clinicopathologic variables of the patient with ILC. Statistical significance was accepted for *P* < 0.05.

## Results

**Figure 1:**
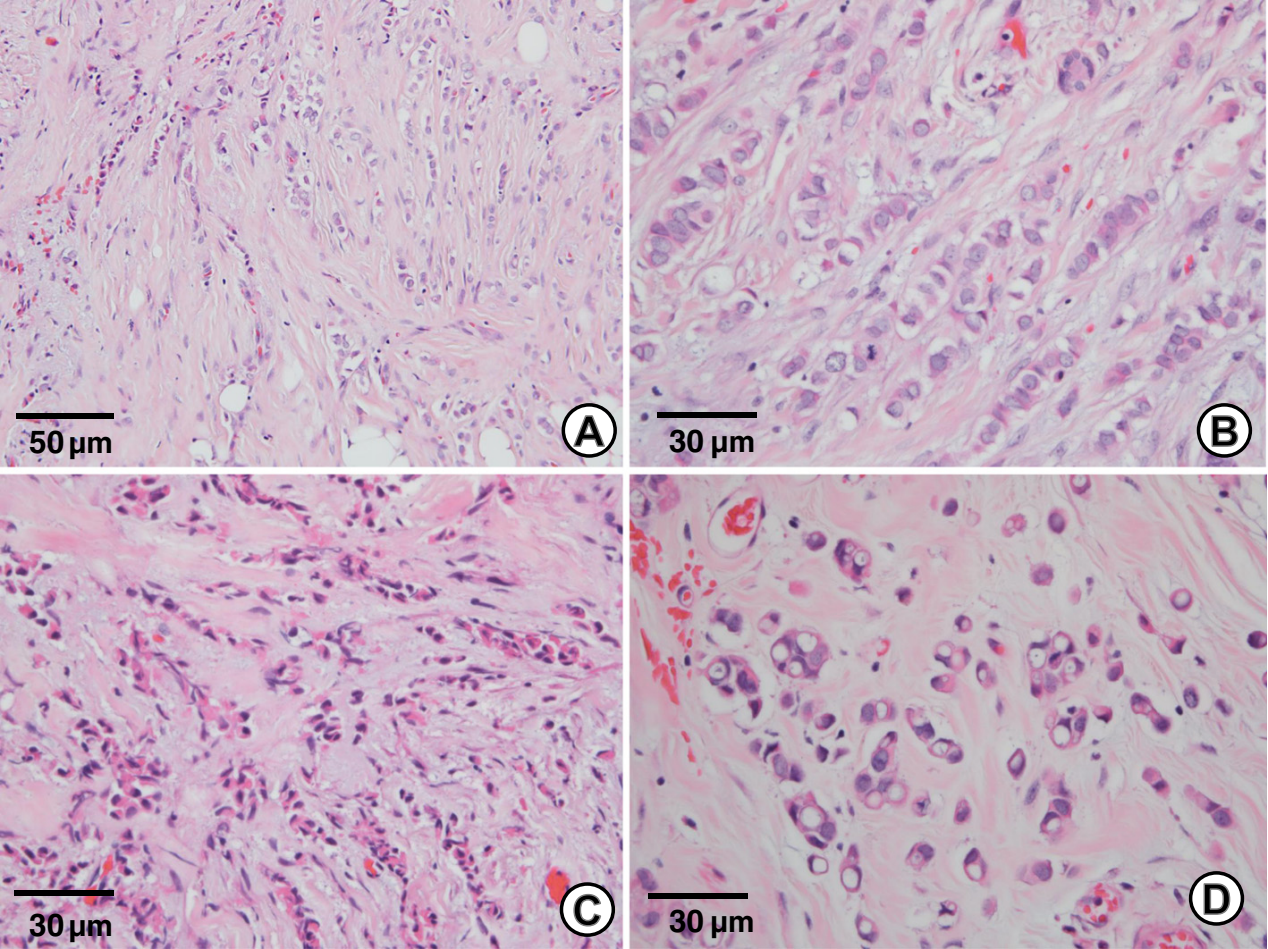
**Classical invasive lobular carcinoma. (A)** ILC shows a single file pattern in a dense fibrous stroma with two different cell types. (H & E, ×200) **(B)** One cell type had a large round vesicular nucleus with a pale eosinophilic cytoplasm. (H & E, ×400) **(C)** The other cell type had a small hyperchromatic nucleus with dense eosinophilic cytoplasm. (H & E, ×400) **(D)** Cells with intracytoplasmic lumens resembling signet ring cells. (H & E, ×400).

A total of 54 cases of ILC demonstrated morphologic features of ILCs, including discohesive cells with a linear or nonlinear growth pattern (Figure 1A). The ILC did not always have the typical uniform bland cells. The cells had large, round, vesicular nuclei surrounded by a thin rim of cytoplasm (Figure 1B) and small hyperchromatic nuclei (Figure 1C) or intracytoplasmic lumina (Figure 1D). The lobular phenotype of all cases was confirmed by the loss of membranous E-cadherin staining in the tumor cells. Expression of EZH2 was analyzed by immunohistochemical staining. In total, 49 cases were scored for expression of EZH2 and the remaining cases were excluded from further analyses because of insufficient tumor size or due to artifacts. Three cases were associated with synchronous or meta-synchronous ILC at the time of diagnosis or during the follow-up period.

### The association between EZH2 expression and clinicopathologic characteristics of ILC

The clinicopathologic characteristics of classical-type ILC are shown in Table 1. No statistically significant difference was observed for most variables, including age, menstrual status, body mass index, multifocality, tumor stage (pT), lymph node stage (pN), estrogen receptor, and progesterone receptor but the difference between EZH2 expression and higher nuclear grade was statistically significant.

**Table 1:** Clinicopathologic characteristics of ILC in this study

		**EZH2 expression *N *** **(%)**				***P***
	**Total *N *** **(%)**	**0% to 5%**	**5% to 25%**	**25% to 50%**	**>50%**	
Age at diagnosis						0.255
<50	28 (57.1)	3 (37.5)	6 (75.0)	2 (33.3)	17 (63.0)	
≤50	21 (42.9)	5 (62.5)	2 (25.0)	4 (66.7)	10 (37.0)	
Menstrual status						0.472
Premenopausal	27 (55.1)	4 (50.0)	6 (75.0)	2 (33.3)	15 (55.6)	
Postmenopausal	22 (44.9)	4 (50.0)	2 (25.0)	4 (66.7)	12 (44.4)	
Body mass index						0.647
Underweight (<20)	3 (6.1)	1 (12.5)	0 (0)	1 (16.7)	1 (3.7)	
Normal (≥20, <25)	29 (59.2)	4 (50.0)	6 (75.0)	2 (33.3)	17 (63.0)	
Overweight (≥25)	17 (34.7)	3 (37.5)	2 (25.0)	3 (50.0)	9 (33.3)	
Side of primary tumor						0.587
Left	25 (51.0)	5 (62.5)	4 (50.0)	4 (66.7)	12 (44.4)	
Right	21 (42.9)	3 (37.5)	3 (37.5)	1 (16.7)	14 (51.9)	
Bilateral	3 (6.1)	0 (0)	1 (12.5)	1 (16.7)	1 (3.7)	
Multifocality						0.981
Monofocal	38 (77.6)	6 (75.0)	6 (75.0)	5 (83.3)	21 (77.8)	
Multifocal	11 (22.4)	2 (25.0)	2 (25.0)	1 (16.7)	6 (22.2)	
T stage						0.770
T1	22 (44.9)	4 (50.0)	5 (62.5)	2 (33.3)	11 (40.7)	
T2	23 (46.9)	3 (37.5)	3 (37.5)	4 (66.7)	13 (48.1)	
T3	4 (8.2)	1 (12.5)	0 (0)	0 (0)	3 (11.1)	
Lymph node metastasis						0.403
N0	28 (57.1)	6 (75.0)	6 (75.0)	4 (66.7)	12 (44.4)	
N1	8 (16.3)	1 (12.5)	2 (25.0)	0 (0)	5 (18.5)	
N2	4 (8.2)	1 (12.5)	0 (0)	0 (0)	3 (11.1)	
N3	9 (18.4)	0 (0)	0 (0)	2 (33.3)	7 (25.9)	
Nuclear grade						0.025
1	7 (14.3)	4 (50.0)	2 (25.0)	0 (0)	1 (3.7)	
2	32 (65.3)	4 (50.0)	5 (62.5)	5 (83.3)	18 (66.7)	
3	10 (20.4)	0 (0)	1 (12.5)	1 (16.7)	8 (29.6)	
Estrogen receptor status						0.629
Negative	13 (26.5)	1 (12.5)	3 (37.5)	1 (16.7)	8 (29.6)	
Positive	36 (73.5)	7 (87.5)	5 (62.5)	5 (83.3)	19 (70.4)	
Progesterone receptor status						0.615
Negative	10 (20.4)	2 (25.0)	2 (25.0)	0 (0)	6 (22.2)	
Positive	39 (79.6)	6 (75.0)	6 (75.0)	6 (100.0)	21 (77.8)	
Cerb-B2 status						0.410
Negative	39 (79.6)	5 (62.5)	7 (87.5)	4 (66.7)	23 (85.2)	
Positive	10 (20.4)	3 (37.5)	1 (12.5)	2 (33.3)	4 (14.8)	
p53						0.093
Negative	37 (75.5)	4 (50.0)	5 (62.5)	4 (66.7)	24 (88.9)	
Positive	12 (24.5)	4 (50.0)	3 (37.5)	2 (33.3)	3 (11.1)	

### The association between EZH2 expression and nuclear grade of ILC

**Figure 2:**
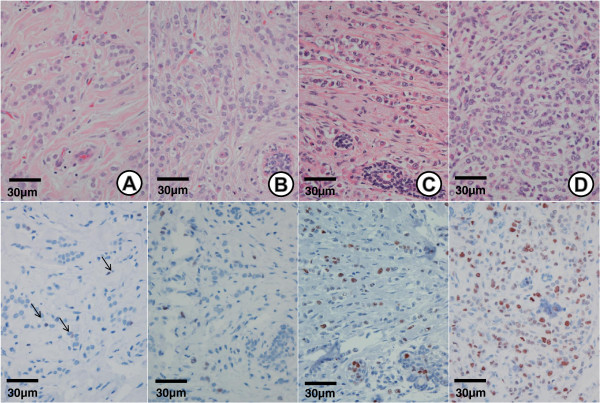
**EZH2 expression in invasive lobular carcinoma.** H & E staining (upper panel, ×400) and EZH2 staining (lower panel, ×400) of ILC. **(A)** Nuclear grade 1 showing <5% nuclear EZH2 expression. **(B)** Nuclear grade 2 showing >5 to 25% nuclear EZH2 expression. **(C)** Nuclear grade 3 showing >25 to 50% nuclear EZH2 expression. **(D)** Nuclear grade 3 showing >50% nuclear EZH2 expression. (Each upper and lower panel was taken from a representative same tumor).

**Figure 3:**
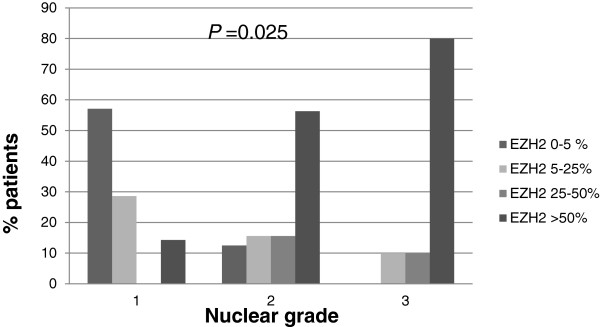
EZH2 distribution differs by nuclear grade of patients with invasive lobular carcinoma.

All cases were morphologically consistent with the lobular carcinoma growth pattern and had a score of 3 for tubule formation and a score of 1 for mitosis. The nuclear scores were 1 (7/49 (14.3%)), 2 (32/49 (65.3%)), and 3 (10/49 (20.4%)). Nuclear grade 1 had four cases (57%) of EZH2 score 1 (Figure 2A), two (29%) of EZH2 score 2, and one (14%) of EZH2 score 4. Nuclear grade 2 had 4 cases (12%) of EZH2 score 1, 5 (16%) of EZH2 score 2 (Figure 2B), 5 (16%) of EZH2 score 3, and 18 (56%) of EZH2 score 4. Nuclear grade 3 had two cases (22%) of EZH2 score 3 (Figure 2C), and seven (78%) of EZH2 score 4 (Figure 2D). In summary, 80% of nuclear grade 3 was associated with EZH2 score 4 and 86% of nuclear grade 1 had an EZH2 score of 1 or 2. Nuclear grade 2 showed relatively varied distributions of EZH2 expression, but 56.3% had an EZH2 score of 4. However, the percentage of cases with an EZH2 score of 4 was similar to that of cases with EZH2 scores of 1 to 3, when based on 50% of EZH2 expression (Figure 3).

The majority of the ILCs were grade 2 in the Nottingham system; 7 cases had a histologic grade of 1 and 42 cases were histologic grade 2. The histologic grade of our cases was dependent on the nuclear grade.

### EZH2 expression in benign and malignant lesions of ILC

Tumor cells of all cases exhibited EZH2 nuclear staining, but the proportion of EZH2 expression varied. Twenty-six (52%) cases exhibited >50% EZH2-positive nuclei, 5 (10%) 25 to 50%, 10 (20%) 5 to 5%, and 8 (18%) 0 to 5% EZH2-positive nuclei. Of the 49 cases, 17 (34.7%) were associated with classical lobular carcinoma *in situ*, 6 (32%) cases exhibited >50% EZH2-positive nuclei of both lobular carcinoma *in situ* and ILC, but 2 (12%) cases exhibited completely EZH2-negative nuclei in lobular carcinoma *in situ* and <5% EZH2-positive nuclei in ILC.

EZH2 expression was also found in both intraductal hyperplasia and non-proliferative glandular epithelium. EZH2 expression in intraductal hyperplasia was positive both outside and within the ILC, but the EZH2 expression in non-proliferative glandular epithelium varied and was negative within the tumor or positive apart from the ILC. Non-proliferative glandular epithelium near intraductal hyperplasia tended to be positive for EZH2.

## Discussion

Breast cancer represents a heterogenous group. ILC is the second most common subtype and has a typical uniform microscopic pattern. However, ILC does not always have the typical uniform bland cell microscopic pattern and can be divided into the classic form and variants. Depending on the histologic or cytologic findings, variants include the alveolar, solid, pleomorphic, signet ring cell, histiocytoid, and apocrine subtypes [12].

E-cadherin immunostaining has been used to distinguish lobular and ductal carcinoma in ambiguous cases because ILC is characterized by a decrease in membrane E-cadherin immunohistochemical reactivity when compared with invasive ductal carcinoma. E-cadherin is a key component of the adherens junctions, which are structures that play crucial roles in the maintenance of epithelial integrity. In cancer, the loss of E-cadherin function through genetic or epigenetic mechanisms has been implicated in the progression and metastasis of numerous malignancies [13].

Based on our study of the literature, we recognized that EZH2 expression has two implications in breast cancer. One is that EZH2 overexpression in cancer is consistently associated with poor prognosis and aggressive behavior, including invasiveness and metastatic potential [14]. The other is that EZH2 overexpression in normal epithelium predicts a progression to malignancy and is indicative of a precancerous state [10]. It is postulated that EZH2 promotes breast cancer progression by transcriptional repression of tumor suppressors and by maintaining the cells in a stem cells state [15].

The three strongest prognostic factors in operable breast cancer are primary tumor size, lymph node status, and tumor grade. The most commonly used grading system for breast cancer is the Nottingham combined histologic grade (Elston-Ellis modification of Scarff-Broom-Richardson grading system), also known as the Nottingham grading system, which is based on tubule formation, nuclear pleomorphism, and mitotic count [16]. Although the prognostic significance of histologic grade has been assessed in ductal carcinoma, its value for ILC is still controversial because ILC is histologically characterized by the absence of tubule formation (except for the tubulolobular variant), relatively uniform cells, and low mitotic counts [17], and some studies have reported a limited prognostic value of grading [18].

EZH2 regulates several genes associated with cell proliferation (RAD51, RUNX3, CDKN1C (p57KIP2), and so on) and invasion (FOXC1 and CDH1 (E-cadherin)), so increased EZH2 reduces CDH1 (E-cadherin) expression [19]. We proposed that all ILC and lobular carcinomas *in situ* showed immunohistochemically EZH2 overexpression because most of them have lost the expression of E-cadherin. However, our results show that some cases of ILC showed EZH2 overexpression in various proportions and intensities. So we think that increased EZH2 expression may not completely inhibit CDH1 (E-cadherin) expression. Yoo *et al*. [19] reported that increased histone deacetylase levels are a prerequisite for EZH2-controlled histone methylation and so in EZH2 overexpression in breast epithelial cells, elevated expression of EZH2 is not sufficient to increase enzymatic activity for H3K27me3 at the CDH1 promoter.

In our study, we investigated the association between EZH2 expression and clinicopathologic factors in ILC. We found that EZH2 expression was associated with a high nuclear grade. We defined ILC cells as discohesive cells with a linear or nonlinear growth pattern with E-cadherin loss on immunohistochemical staining. All cases had no tubule formation and fewer than two mitoses per ten high-power fields. The nuclear scores were score 1 (7 cases), score 2 (32 cases) and score 3 (10 cases). A total of 7 cases (14.3%) were histologic grade 1 and 42 cases (85.7%) were histologic grade 2 in the Nottingham system and 32 cases of nuclear grade 2 included 18 cases (56.3%) that expressed more than 50% EZH2-positive and 14 cases (43.8%) expressed less than 50% EZH2-positive. The ten cases of nuclear grade 3 included eight cases (80%) that expressed more than 50% EZH2-positive. Rakha *et al*. [17] reported that the majority of ILC cases showed a moderate degree of cellular or nuclear pleomorphism and low mitotic counts and that the prognostic significance of histologic grading was well demonstrated by the Nottingham grading system. The authors suggested an alternative, simpler system, such as combined pleomorphism and mitosis only (by exclusion of tubule formation or by splitting grade 2 tumors into two subgroups). If grade 2 tumors are divided into two subgroups, our opinion is that nuclear grade and EZH2 expression should be included in the criteria. Orvieto *et al*. [12] reported that although variants of ILC did not have the independent prognostic value, histopathologic subtyping of ILC was clinically useful, and provided additional information. In the recent literature, the pleomorphic variant has been considered as a particularly aggressive variant of ILC [20]. Therefore, histological and cytological variations are present that suggest changes in the nature of the lesion. It is important to diagnose variants of ILC with a consensus on the definition of the variants.

Women with intraductal hyperplasia have a greater risk of developing breast cancer than do women without this abnormality. Benign lesions containing genetic and epigenetic alterations are generally accepted to render women possibly more susceptible to neoplastic transformations. The development of carcinomas is preceded by an expansion of cells with the ability to initiate preneoplastic progression [21], but no method exists for identifying these cells. Until now, the earliest recognizable precursor of carcinoma is atypical ductal hyperplasia; the diagnosis is based on pathologic criteria, including cytological and morphological abnormalities. Ding *et al*. [11] reported that EZH2 detection in tissue by immunohistochemistry could potentially constitute the basis for a diagnostic test that distinguishes histologically normal breast tissues at higher risk for neoplastic progression. Yoo *et al*. [19] reported that EZH2 appears to regulate breast tumor initiating cells. In our study, EZH2 expression was found in both intraductal epithelial hyperplasia and in histologically normal epithelium. Comparing the intensity and percentage of EZH2 positive cells, no significant difference was found between the intraductal hyperplasia and the tumor, and the intraductal hyperplasia showed EZH2 expression regardless of location. However, histologically normal epithelium showed weaker intensity and a lower percentage of EZH2 expression than did tumor tissue. Entrapped normal glands showed completely negative staining for EZH2.

## Conclusions

In conclusion, the prognostic value of the histologic grade of ILC, as assessed by the Nottingham grading system, is controversial. Variants of ILC could affect good or poor prognosis; no precise consensus has been established to define the variants of ILC. Our findings indicate that EZH2 expression is associated with a high nuclear grade and that most ILCs have histologic grade 2 with nuclear grade 2 or 3. Therefore, our opinion is that if ILC is diagnosed by separating the classic type and variants and if both EZH2 expression and nuclear grade are considered, the Nottingham grading system would be a more accurate prognostic factor.

## Abbreviations

EZH2: Enhancer of Zeste Homolog 2; H & E: Hematoxylin and Eosin; ILC: Invasive lobular carcinoma; pN: Lymph node stage; PRC2: Polycomb repressive complex 2; pT: Tumor stage.

## Competing interests

No potential conflict of interest relevant to this article was reported.

## Authors’ contributions

EJC organized all of the study. SYP, HSK, JSS participated in the study design and revised the manuscript. SGR collected and analysed the clinicopathologic data. All authors read and approved the final manuscript.
